# Hemophilia A developing cerebral infarction after surgical treatment of giant hemophilic pseudotumor: a case report

**DOI:** 10.1186/s12893-022-01753-2

**Published:** 2022-08-06

**Authors:** Yiqing Ling, Zhenyu Shi, Chenying Su, Xiaochen Liu, Lingxin Zheng, Xiaohao Pan, Yan Sun, Xuan Zhang, Jinling Wei, Ju Li, Peijian Tong, Taotao Xu

**Affiliations:** 1 The First Affiliated Hospital of Zhejiang, Chinese Medical University, You Dian Road No.54, Hangzhou, 310006 Zhejiang Province People’s Republic of China; 2 The First Affiliated Hospital of Wenzhou Medical University, Wenzhou, 325000 Zhejiang People’s Republic of China; 3 University of Toledo Medical Center, Toledo, OH 43614 USA; 4 Zhejiang Chinese Medical University, Hangzhou, 310053 Zhejiang People’s Republic of China; 5 Tongde Hospital of Zhejiang Province, Hangzhou, 310007 Zhejiang People’s Republic of China

**Keywords:** Hemophilia A, Factor VIII deficiency, Cerebral infarction, Hemophilic pseudotumor, Case report

## Abstract

**Background:**

Cerebral infarction (CI) is an unusual complication in patients with bleeding disorders. To our knowledge, this is the first case of postoperative internal border-zone infarction (I-BZI) from Hemophilia A.

**Case presentation:**

We present a case of Hemophilia A developing I-BZI, after surgical treatment of giant hemophilic pseudotumor. A 36-year-old man was introduced from other hospital by Hemophilia with giant hemophilic pseudotumor in his left thigh. Patient and his relatives did not have a history of thrombophilia. After excluding the relevant surgical contraindications, we performed the operation of pseudotumor resection. Prior to surgery, blood tests revealed hemoglobin of 137 g/L. FVIII activity was 1.5%. Activated partial thromboplastin time (APTT) was 71.50 s and D-dimer was 3.33 mg/L FEU. Immediately before surgery, the patient received an intravenous infusion of FVIII products (Xyntha^®^) at a dose of 3500 IU for his body weight of 80 kg. Post-operative day two (POD2), patient developed vomiting, decreased response, and dysarthria. Hemoglobin was 54 g/L with blood pressure of 110/70 mmHg. Magnetic resonance imaging of the brain showed there were multiple acute cerebral infarctions in bilateral lateral ventricles (internal border zone) and multiple ischemic foci in the white matter areas and basal ganglia of the bilateral cerebral hemispheres. This case suggested that acute severe anemia can be one of the causes of I-BZI.

**Conclusions:**

For the treatment of I-BZI caused by acute anemia from Hemophilia A, volume expansion, red blood cell supplement and continuous improvement of coagulation with suitable dose of factor VIII (FVIII) should be considered to improve prognosis.

## Background

Hemophilia is an X-linked recessive congenital bleeding disorder characterized by the absence or deficiency in factor VIII/IX pro-coagulant function [1]. The majority of bleeding episodes in hemophilic patients occur within the musculoskeletal system, mainly in the joints [2]. However, approximately 30% of cases involve the muscles, especially muscles with a rich blood supply [3, 4]. Recurrent bleeding in the muscles may lead to the complication of pseudotumor, which has a reported incidence of 1%-2% in patients with severe coagulation factor deficiency [5]. A hemophilic pseudotumor, also called a hemophilic cyst, is characterized by an encapsulated and slowly expanding hematoma, which may enlarge progressively and become life-threatening [6]. Surgical resection, embolisation or bone graft to the emptied area followed by rational factor replacement therapy is effective in treating hemophilic pseudotumor. Presently, there are few reports of ischemic cerebral infarction (CI), especially internal border-zone infarction (I-BZI) in patients with Hemophilia [7]. More importantly, the etiology of the ischemia needed to be identified as it will govern the appropriate treatment. In this case report, we present a case of Hemophilia A developing cerebral infarction after surgical treatment of giant hemophilic pseudotumor.

## Case report

**Fig. 1:**
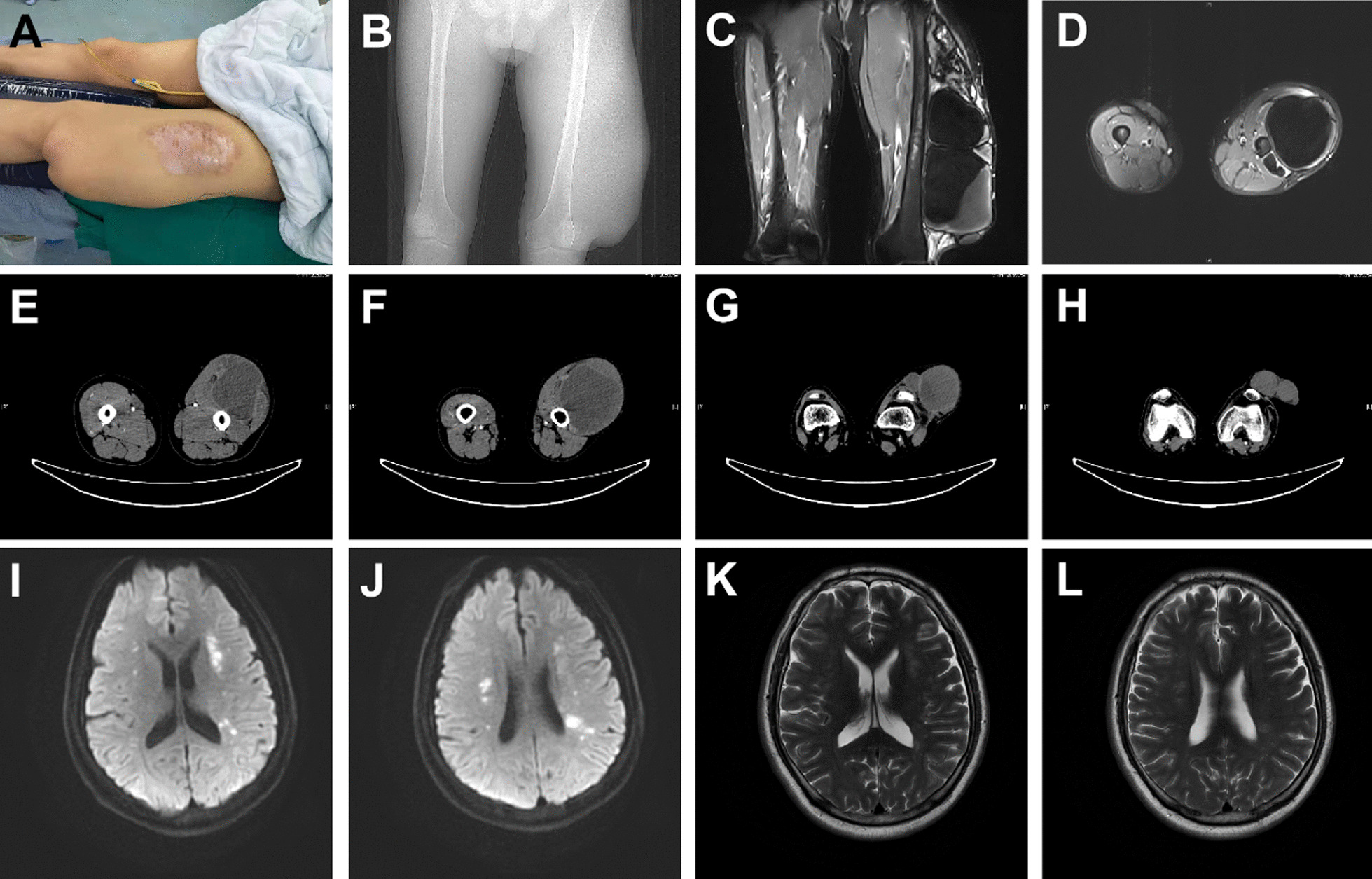
Preoperative photograph of left thigh showing a giant mass on the anterolateral region of left thigh (**A**). Anteroposterior radiograph of the left thigh showing a giant soft-tissue mass located at the left thigh without periosteal reaction of femur shaft (**B**). Coronal and axial Magnetic resonance imaging (MRI) of the left thigh showing the large pseudotumor masses on the anterolateral region of left thigh (**C**, **D**). Axial computerized tomography of leg showed intramuscular hematoma in left thigh (**E**–**H**). Diffusion weighted MRI and T2 weighted MRI of the brain showed there were multiple acute cerebral infarctions in bilateral lateral ventricles (internal border zone) and multiple ischemic foci in the white matter areas and basal ganglia of the bilateral cerebral hemispheres (**I**–**L**)

A 36-year-old male with severe Hemophilia A presented with a mass in his left thigh. The mass was noticed 10 years ago at which time patient declined intervention. Patient had inconsistent factor VIII (FVIII) treatment since patient was 12 years old. Patient and his relatives did not have a history of thrombophilia. In Jan 2021, the patient was admitted for swelling in the left thigh. Physical examination on admission showed flexion contracture of the left knee due to severe pain and a hard mass over the anterolateral left thigh with pressure pain (Fig. 1A). Neurologic exam of the lower extremity was normal. There were symmetrical and strong pulses in bilateral dorsalis pedis artery on palpation. Radiographs, CT, and MRI showed a 45 cm × 13 cm × 12 cm pseudotumor in the anterolateral left thigh with multiple large irregular cystic lesions. There was no cortical destruction of the left femur (Fig. 1B–H). A focal ultrasound revealed a giant intramuscular pseudotumor with irregular borders and mass effect on adjacent structures. No definite feeding artery was identified. The neighboring blood vessel around the mass was difficult to detect via the vascular ultrasound due to the shadowing from the giant mass. However, an undisturbed blood flow was seen in the distal portion of the pseudotumor. Blood tests on 01/26/2021 revealed hemoglobin of 137 g/L. FVIII activity was 1.5%. Activated partial thromboplastin time (APTT) was 71.50 s and D-dimer was 3.33 mg/L FEU (Table 1). The FVIII inhibitor test and human immunodeficiency virus (HIV) screening was negative.

**Table 1:** Summary of laboratory analysis and coagulation factor administration at different time point in this study

Date	Hemoglobin (g/L)	Platelet (10^9^/L)	FVIII activity (%)	APTT (s)	D-dimer (mg/L FEU)	FIB (g/L)	Blood pressure (mmHg)	Volume of coagulation factor administration
01/26/2021 (on admission)	137	204	1.5	71.50	3.33	3.11	145/96	–
01/28/2021 (prior to surgery)	–	223	50.4	43.90	4.48	2.94	112/63	Bolus dose of 3500 IU + 1000 IU
01/29/2021 (POD1)	83	180	29.8	47.10	1.06	2.74	134/75	Maintenance dose of 2000 IU q8h
01/30/2021 (POD2: patient with slurred speech and decreased responsiveness)	54	173	17.7	52.70	0.71	4.77	110/70	Bolus dose of 4000 IU and maintenance dose of 4000 IU q8h
01/30/2021 (after intravenous injection with RBC 2U and plasma 390 ml)	62	176	43.5	40.60	0.66	4.53	115/72	Maintenance dose of 4000 IU q8h
01/31/2021 (POD4: patient with worsening alteration of consciousness)	47	157	76.0	46.40	0.66	3.83	120/70	Maintenance dose of 4000 IU q8h
01/31/2021 (after intravenous injection with RBC 3U and plasma 350 ml)	73	213	96.2	38.90	0.46	6.65	140/80	Maintenance dose of 4000 IU q8h

The patient underwent pseudotumor resection under general anesthesia on 01/28/2021. Immediately prior to surgery, the patient received an intravenous infusion of FVIII products (Xyntha^®^) at a dose of 3500 IU for his body weight of 80 kg. According to his FVIII activity (Table 1), we appropriately adjusted the volume of coagulation factor administration. Intracapsular resection of the pseudotumor was performed because of the ill-defined borders and unknown malignant potential of the mass. Intra-operative dissection of the pseudotumor revealed several liters of brown-greyish fluid. Ossified hematoma and old blood clots were found within the pseudotumor capsule. The resected mass weighed 2000 g. The resection cavity was subsequently irrigated with dilute hydrogen peroxide solution and betadine. After which the cavity was packed with gelatin sponge and the dead space was closed using overlapping sutures. Total blood loss during surgery was 900 ml, and 2 U of red blood cell were transfused.

On 01/29/2021, the patient’s vital signs were stable. The left thigh was slightly swollen. Bloody liquid of 110 ml was drawn from the drainage tube, and the incision was dry and clean. Coagulation factor VIII was changed to maintain at a dose of 2000 IU q8h. The result of hemoglobin, FVIII activity, APTT and D-dimer are summarized on Table 1. Until the night of 01/30/2021, patient had two episodes of vomiting and developed slurred speech with decreased responsiveness. Heart rate on ECG was 110 bpm with blood pressure about 110/70 mmHg, and oxygen saturation was 98–100% under oxygen inhalation (3 L/min). Physical exam revealed that patient was aphasic but was able to follow commands. Upper extremities were within normal limits. The abdomen was soft and nontender. The right lower extremity was normal. The left thigh was swollen, and the left leg had pitting edema. Patient was not able to move it upon command. Bilateral Babinski’s signs were positive. Blood work showed hemoglobin was 54 g/L, FVIII activity was 17.7%, APTT was 52.70 s, and D-dimer was 0.71 mg/L FEU (Table 1). Craniocerebral CT scan showed no obvious abnormal changes in brain parenchyma. After intravenous injection of 4000 IU coagulation factor, 4000 IU q8h maintained, and RBC 2 U and plasma 390 ml were infused. Blood work showed hemoglobin was 62 g/L, FVIII activity was 43.5%, APTT was 40.60 s, and D-dimer was 0.66 mg/L FEU (Table 1). In the morning of 01/31/2021, the patient’s continued to have worsening alteration of consciousness. Blood work showed hemoglobin was 47 g/L, FVIII activity was 76.0%, APTT was 46.40 s, and D-dimer was 0.66 mg/L FEU (Table 1). Blood gas analysis showed that pH was 7.460, partial pressure of oxygen was 152.0 mmHg, partial pressure of carbon dioxide was 36.8 mmHg, and lactic acid was 0.90 mmol/L. The inhibitor test revealed FVIII inhibitor < 0.60BU. After intravenous injection of RBC 3U, plasma 350 ml were infused. Blood work showed the hemoglobin was to 73 g/L, FVIII activity was 96.2%, APTT was 38.90 s, and D-dimer was 0.46 mg/L FEU (Table 1). Magnetic resonance imaging of the brain showed there were multiple acute cerebral infarctions in bilateral lateral ventricles (internal border zone) and multiple ischemic foci in the white matter areas and basal ganglia of the bilateral cerebral hemispheres (Fig. 1I–L).

After multidisciplinary consultation, supportive treatment with RBC and plasma infusion and coagulation factor administration continued. On 02/02/2021, patient started to gradually recover starting with single syllable and continued with double syllables the next day. One week after the initially neurologic deficit, the patient was at near baseline consciousness and normal speech.

## Discussion

Border zone infarction (BZI) occur at the anastomotic watershed areas of two main arteries [8]. This type of infarction accounts for about 10% of all CI [9]. The pathophysiology is thought to be ischemic damage due to hypoperfusion of the distal vascular territory. BZI was divided into cortical border-zone infarction (C-BZI) or internal border-zone infarction (I-BZI). C-BZI is associated with embolic pathogenesis and less frequently due to hemodynamic compromise. I-BZI is from hypoperfusion caused mainly by arterial stenosis, occlusion, or hemodynamic compromise [10, 11]. The internal border-zone is supplied by the superficial and deep perforating arteries of the middle cerebral artery (MCA) and the anterior cerebral artery (ACA) [10]. In general, hemodynamic compromise is considered as hypotension in patients with severe arterial stenosis or acute hypotension [12]. However, Amin-Hanjani et al. found that the hypoperfusion symptoms alone correlated poorly with actual hemodynamic compromise as assessed by quantitative magnetic resonance angiography [13].

Recently, researchers have noticed and revealed the potential correlations among bleeding, anemia, and CI [14, 15]. Previous research has considered that bleeding and subsequent anemia to be a precipitant for CI and proposed that the hemodynamic compromise and enhanced thrombosis were the mainly pathogenic mechanisms. In addition, Tsai et al. reported 12 patients with acute anemia in the case of acute blood loss, without systemic hypotension [16]. The study found that all bilateral BZI had I-BZI thought to be because acute anemia led to cerebral blood flow insufficiency and oxygen carrying capacity reduction resulting in CI when hemoglobin levels drop below the critical level, especially in patients with intracranial stenosis. In our case, no clear embolic source was found. When the patient was considered to have bilateral I-BZI, that is, at the beginning of neurological deficit (110/70mmhg), there was no systemic hypotension. Patient refused to undergo cerebral magnetic resonance angiography due to rapid recovery and financial reasons. Ambulatory electrocardiogram showed no atrial fibrillation, and echocardiography showed no atrial fibrillation and mural thrombus. No obvious plaque is found in carotid artery by B-ultrasound, so thrombembolia is not considered as the cause of I-BZI. Taking into account of the watershed location, patient’s young age, and his rapid recovery, the root cause was thought to be due to postoperative acute blood loss secondary to Hemophilia A. And while the blood pressure did not decrease significantly, there were signs of acute anemia (hemoglobin 54 g/L). This resulted in ineffective perfusion and decreased oxygen carrying capacity leading to tissue hypoxia and hemodynamic damage. Miyazaki et al. presented the first case of acquired Hemophilia A patient with pseudotumor and I-BZI [7]. The patient was not treated surgically, and laboratory tests showed severe anemia (hemoglobin of 5.5 g/dL), APTT of 99.4 s. In our case, preoperative hemoglobin of the patient was 137 g/L, whereas the postoperative hemoglobin dropped to 54 g/L. Postoperative day one, hemoglobin continued to drop 47 g/L. In addition, while hemophilic pseudotumor is a slow process, I-BZI occurs in acute or chronic anemia as shown by both Masayuki and our report. On the other hand, several studies have documented that severe or chronic iron-deficiency anemia patients were accompanied by reactive thrombocytosis, which was considered as a risk factor for carotid artery thrombus formation and might lead to CI [14, 17]. However, Tsai et al. considered that reactive thrombocytosis was not a possible contributing factor to CI in patients with acute anemia because thrombocytosis was not present at all in any of patients with acute anemia and CI [16]. In our case, on POD2 and POD4, the levels of APTT, D-dimer and FIB were mildly elevated, while the hemoglobin was 54 g/L and 47 g/L, respectively, and platelets were within normal range (Table 1), indicating acute anemia contributes to CI via the decrease of oxygen carrying capacity rather than the change of coagulation and fibrinolysis. Nevertheless, the definite mechanisms of their associations between CI and acute anemia require investigation in future studies.

In the treatment of cerebral ischemia, the etiology of the ischemia is vital to determining the treatment options. Our patient with Hemophilia A was high risk of massive bleeding from the surgical intervention. The combination of repetitive hemorrhage and acute blood loss postoperatively resulted in the watershed infarction of bilateral lateral ventricles and multiple ischemic foci in the white matter areas and basal ganglia of the bilateral cerebral hemispheres. Although the treatment for thrombotic and stenotic ischemia is thrombectomy or thrombolysis, it is essential to urgent blood transfusion and rapid correction of the underlying cause of acute bleeding. Volume repletion and expansion is needed to address the hypoperfusion. Especially for patients with acute anemia, only expansion is not enough, but also the supplement of red blood cells is needed to increase oxygen carrying capacity and improve ineffective perfusion. On the other hand, improvement of the patient’s coagulation function was essential for the underlying Hemophilia A, while this strategy seems to be paradoxical to CI. It is worth mentioning that prothrombin complex is one of the commonly used coagulation drugs in Hemophilia patients, but some studies have shown that prothrombin complex has been reported to increase the probability of cerebral infarction [18]. Accordingly, as to I-BZI caused by acute anemia in hemophilic patients, the maintenance infusion with FVIII seems to be recommended. Admittedly, the present study has a limited number of patients with I-BZI caused acute anemia, and the preliminary results should be interpreted cautiously. More case collection and awareness are required to clarify how to rapid correction of the underlying cause of acute bleeding in hemophilic patients.

## Conclusions

I-BZI can occur in acute or chronic anemia if hemoglobin is below a certain level. Further studies will have to be done to determine the threshold. For the treatment of I-BZI caused by acute anemia from Hemophilia A, volume expansion, red blood cell supplement and continuous improvement of coagulation with suitable dose of FVIII should be considered to improve prognosis.

## Data Availability

The original contributions presented in the study are included in the article/supplementary material, further inquiries can be directed to the corresponding author/s.

## Abbreviations

FIB: Fibrinofen
MCA: Middle cerebral artery
ACA: Anterior cerebral artery
BZI: Border-zone infarction
C-BZI: Cortical border-zone infarction
APTT: Activated partial thromboplastin time
HIV: Human immunodeficiency virus
CI: Cerebral infarction
POD2: Post-operative day two
POD4: Post-operative day four
FVIII: Factor VIII
I-BZI: Internal border-zone infarction
RBC: Red blood cell
ECG: Electrocardiogram
